# The regulatory mechanism and therapeutic potential of transcription factor EB in neurodegenerative diseases

**DOI:** 10.1111/cns.13985

**Published:** 2022-10-02

**Authors:** Fengjuan Jiao, Bojie Zhou, Lingyan Meng

**Affiliations:** ^1^ School of Mental Health Jining Medical University Jining China; ^2^ Shandong Key Laboratory of Behavioral Medicine, School of Mental Health Jining Medical University Jining China

**Keywords:** autophagy, autophagy‐lysosomal pathway, neurodegenerative disease, TFEB agonist, transcription factor EB

## Abstract

The autophagy‐lysosomal pathway (ALP) is involved in the degradation of protein aggregates and damaged organelles. Transcription factor EB (TFEB), a major regulator of ALP, has emerged as a leading factor in addressing neurodegenerative disease pathology, including Alzheimer's disease (AD), Parkinson's disease (PD), PolyQ diseases, and Amyotrophic lateral sclerosis (ALS). In this review, we delineate the regulation of TFEB expression and its functions in ALP. Dysfunctions of TFEB and its role in the pathogenesis of several neurodegenerative diseases are reviewed. We summarize the protective effects and molecular mechanisms of some TFEB‐targeted agonists in neurodegenerative diseases. We also offer our perspective on analyzing the pros and cons of these agonists in the treatment of neurodegenerative diseases from the perspective of drug development. More studies on the regulatory mechanisms of TFEB in other biological processes will aid our understanding of the application of TFEB‐targeted therapy in neurodegeneration.

## Graphical abstract

Abnormal activation of TFEB leads to dysfunction of ALP pathway in neurodegenerative diseases. Growing evidence shows that overexpression of TFEB or TFEB‐targeted small molecules promotes the degradation of abnormal aggregation proteins and significantly improves the abnormal behavioral characteristics of animal models. This review mainly summarize the regulatory mechanism of TFEB and its function in neurodegenerative diseases.

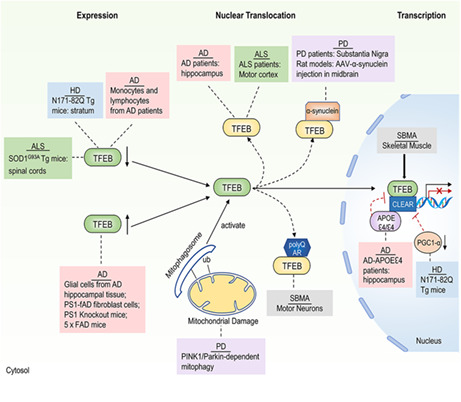

## 1 INTRODUCTION

The accumulation of abnormally assembled intracellular proteins is a common pathological hallmark of neurodegenerative diseases such as Alzheimer's diseases (AD), Parkinson's disease (PD), Huntington's disease (HD) and Amyotrophic lateral sclerosis (ALS), which can cause organelle damage and synaptic dysfunction in the nervous system.^1^, ^2^, ^3^, ^4^ The autophagy‐lysosome pathway (ALP) is one of the main elimination pathways for long‐lived proteins and abnormal organelles in the cytoplasm.^5^, ^6^ In recent years, growing evidence has shown that defects in ALP are strongly associated with neurodegenerative diseases.^7^, ^8^, ^9^, ^10^ As a master regulator of the ALP, TFEB has been widely proven to ameliorate pathology in these diseases when activated.^11^, ^12^, ^13^, ^14^ Moreover, the broad applicability of TFEB and its role in the ALP make it an exceedingly attractive therapeutic target for treating neurodegenerative diseases.

In this review, we briefly introduce the role of TFEB in regulating lysosomal function and autophagy. Furthermore, we provide a description of the mechanism of TFEB activation and the implication of impaired TFEB signaling in neurodegenerative diseases. Finally, the potential therapeutic modulators of TFEB and their applications in neurodegenerative diseases are highlighted.

## 2 MOLECULAR CHARACTERISTICS AND FUNCTIONS OF TFEB

**FIGURE 1:**
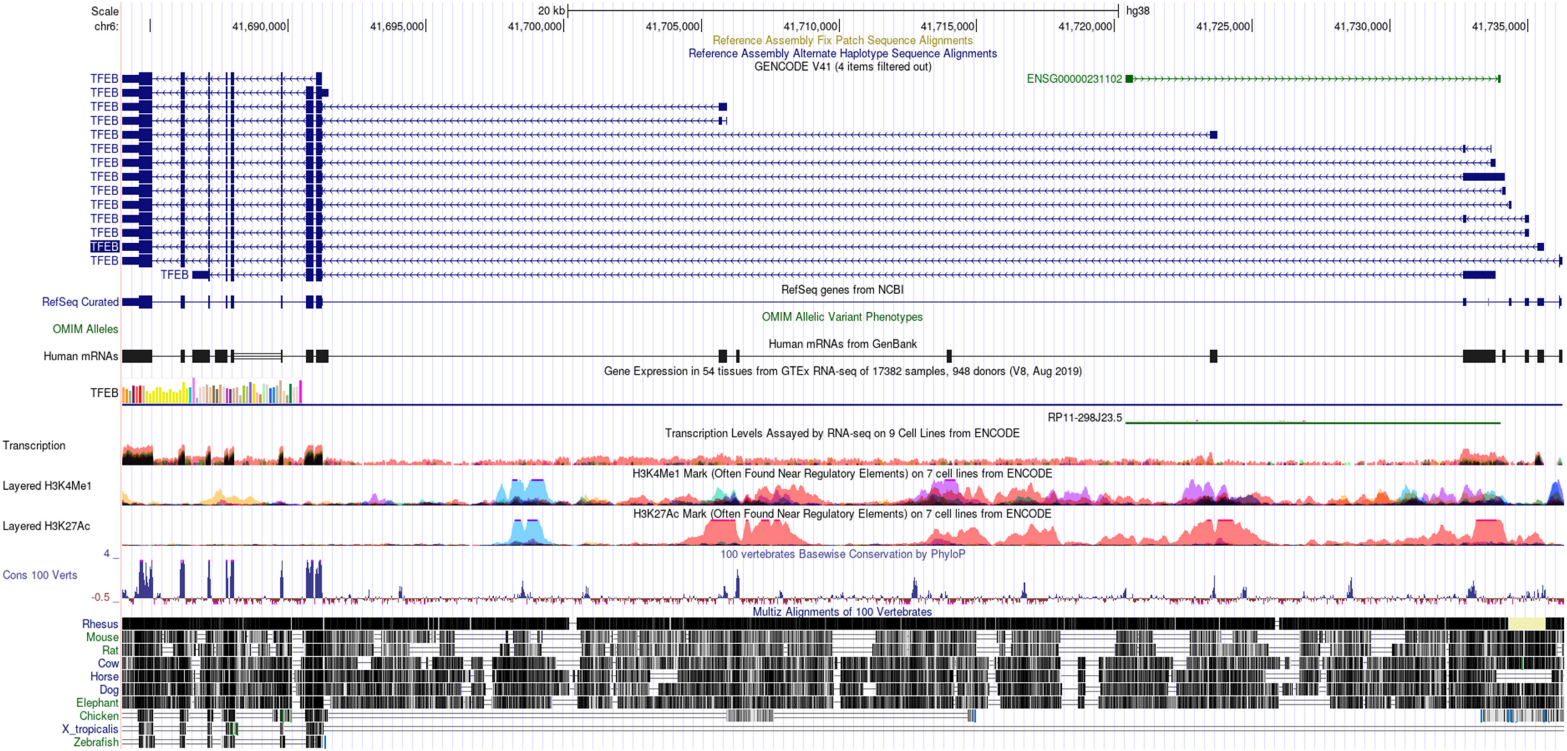
Conservation analysis of TFEB gene. Graphical views showing multi‐species comparisons of TFEB using UCSC genome browser. The conservation scores are indicated by the blue and red peaks.

**FIGURE 2:**
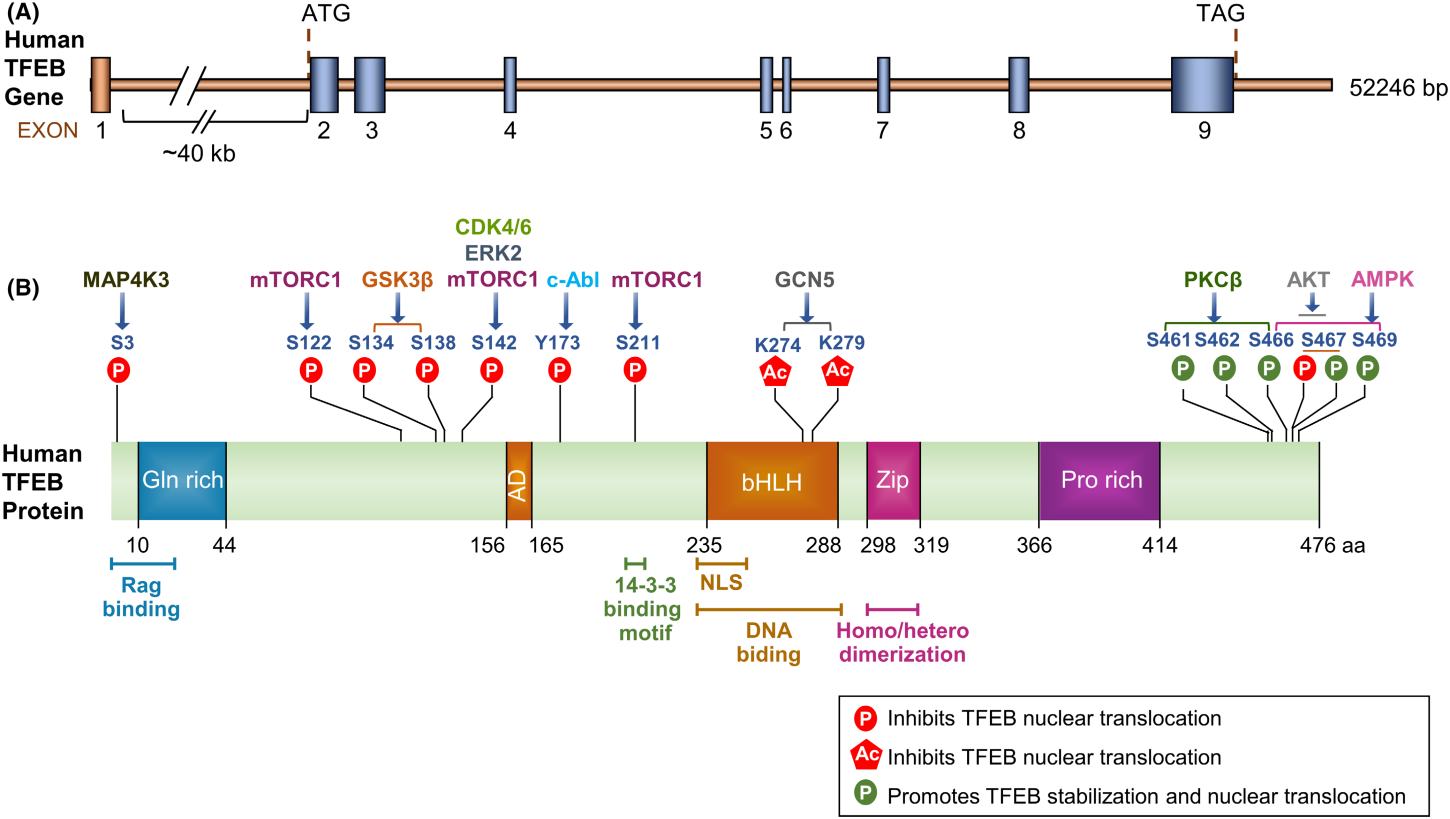
Structure of human TFEB gene and protein. (A) Schematic of human TFEB gene. TFEB gene is composed of nine exons, and ATG start codon and TAG stop codon are located on exon 2 and exon 9, respectively. (B) Domain structure of human TFEB. The N‐terminus of TFEB contains a glutamine‐rich domain, and its C‐terminus contains a proline‐rich domain. The DNA‐binding region is composed of a HLH and a leucine Zip domain. Relevant TFEB phosphorylation and acetylation sites and their regulatory role are indicated in different regions.

TFEB, a member of the microphthalmia transcription factor (MiTF)/TFE family, has been highly conserved during evolution and is widely expressed in multiple species, such as flies, fish, *Caenorhabditis elegans*, birds, and mammals (Figure 1).^15^, ^16^, ^17^, ^18^, ^19^ The human TFEB gene (NC_000006) located on chromosome 6p21.1 is composed of nine exons, with a postulated initiation ATG preceded by a perfect ribosomal binding sequence in exon 2.^20^ This organization generates an mRNA transcript characterized by two non‐coding and eight coding exons. Seven alternative TFEB transcripts containing distinct alternative 5′ exons, which contain the translational start site at exon 2, have been described with differential expression and different tissue distributions (Figure 2A).^20^

TFEB protein has three essential regions similar to other Mif proteins, including DNA‐binding regions, a helix–loop–helix (HLH) and leucine‐zipper (Zip) regions.^21^ The DNA‐binding region is characterized by a HLH and a Zip domain flanked by an upstream basic region that is able to recognize an E‐box sequence (CAYGTG) in the promoter of targeted genes.^22^, ^23^ In addition, the N‐terminus of the TFEB protein contains a glutamine‐rich domain encompassing the binding site of Rag C, while its C‐terminus contains a proline‐rich domain, the function of which has not been reported (Figure 2B).

Lysosomes are key organelles of the cellular degradation and recycling processes, and they are required to maintain cellular homeostasis.^24^ Lysosomes are involved in multiple essential cellular processes, including endocytosis, autophagy, and lysosomal exocytosis.^25^ However, the regulation of lysosome biogenesis and function by cells has remained unanswered for a long time. In 2009, Sardiello, et al. first discovered that lysosomal biogenesis is transcriptionally regulated by a gene network called the coordinated lysosomal expression and regulation (CLEAR) element, a common 10‐base E‐box‐like palindromic sequence, which is regulated by TFEB.^26^ Furthermore, the authors also found that overexpression of TFEB upregulates the expression of genes related to lysosomal enzymes, lysosomal biosynthesis and functions.^26^ TFEB‐mediated activation of the CLEAR network regulates the lysosomal proteostasis by enhancing folding, trafficking and lysosomal activity of a severely destabilized glucocerebrosidase variant (L444P).^27^ Subsequent work has shown that TFEB orchestrates the expression of genes involved in processes such as lysosomal biogenesis, lysosomal exocytosis, endocytosis, membrane repair, and autophagy by direct binding to the CLEAR motif at their promoters.^28^ TFEB was identified as a main player in regulating autophagosome biogenesis and autophagosome‐lysosome fusion by binding to the promoter regions of numerous autophagy genes.^11^ In addition, TFEB enhances the degradation of long‐lived proteins, the clearance of lipid droplets, and damaged mitochondria,^11^, ^29^, ^30^ suggesting that it also plays an important role in modulating the biological process of lipophagy and mitophagy. In addition, TFEB has also been found to regulate lysosomal exocytosis by activating the lysosomal Ca2^+^ channel mucolipin 1 (MCOLN1). This promotes cellular clearance and increases the pool of lysosomes in the proximity of the plasma membrane (PM) to stimulate their fusion to PM.^31^

In a number of disease conditions, the role of TFEB in the control of lysosomal biosynthesis, autophagy, and lysosomal exocytosis was primarily exploited to enhance cellular clearance.^32^ This TFEB‐mediated effect has been widely demonstrated in several cellular and mouse models of human diseases which are characterized by an accumulation of undegraded substances(e.g. AD,^33^, ^34^, ^35^ PD,^36^, ^37^, ^38^, ^39^ HD^40^, ^41^) as well as lysosomal storage diseases (LSD),^27^, ^31^, ^42^ among others.

## 3 THE REGULATION OF TFEB

### 3.1 Transcriptional regulation of TFEB

TFEB expression is transcriptionally regulated by different factors (Table 1). Cyclic adenosine monophosphate (cAMP) response element‐binding protein (CREB), a key transcriptional activator that drives fasting responses, has been identified as a transcriptional activator of TFEB. In addition, CREB upregulates the expression of TFEB and other autophagy genes by recruiting its coactivator, the CREB regulated transcription coactivator 2 (CRTC2), in the liver of fasted mice.^43^ TFEB was also identified as the downstream transcriptional target of a master activator of mitochondrial proliferation. The peroxisome proliferator‐activated receptor γ (PPARγ) coactivator 1α (PGC‐1α) binds to one of the promoters of TFEB, which is essential for TFEB‐mediated mitophagy.^40^, ^44^ In turn, PGC‐1α is a direct target of TFEB, which controls the starvation response by orchestrating PGC‐1α‐PPARα‐mediated lipid catabolism.^30^ Moreover, activated PPARα forms a complex with retinoid X receptor α (RXRα) and PGC‐1α, which is recruited to the TFEB promoter and initiates transcriptional activation of TFEB in brain cells.^45^ Scavenger receptor class B type I (SR‐BI) is an integral membrane glycoprotein, which regulates autophagy, lysosome function, efferocytosis, cell survival, and inflammation.^46^ Furthermore, SR‐BI regulates both basal and inducible expression levels of TFEB by enhancing PPARα activation.^47^

**TABLE 1:** Transcriptional regulation of TFEB

Regulatory factors	Treatment	*Tfeb* mRNA^a^	Mechanisms	References
Transcriptional co‐activators				
CREB	Under nutrient‐deprived conditions	↑	CREB upregulates *Tfeb* by recruiting its coactivator CRTC2	[43]
PGC‐1α	Genetic overexpression of PGC‐1a in HD	↑	PGC‐1α occupies the proximal promoter region for the *Tfeb* isoform with the most 3′ transcription start site	[40]
PPARα	Gemfibrozil; SR‐BI deletion	↑	PPARα recruits RXR, and PGC1 on the PPAR‐binding site on the *Tfeb* promoter; SR‐BI regulates expression levels of TFEB by enhancing PPARα activation	[45, 47]
CARM1	Glucose starvation	↑	CARM1 binds to the transcriptional activation domain of *Tfeb* and increase CLEAR element activity	[58]
YAP	Cardiomyocytes transduced with Ad‐sh‐RagA/B	↑	YAP interacts with TFEB and promotes *Tfeb*‐mediated transcription	[59]
Transcription factors				
TFEB	Starvation	↑	*Tfeb* mediates the positive feedback loop on its own expression by directly binding to its promoter	[30]
Splicing TFEB	Fisetin	↓	Splicing TFEB significant decrease of the promoter activity containing CLEAR	[53]
C‐ETS2	Oxidative stress	↑	By directly binding to *Tfeb* promoter	[48]
FoxO1	Differentiation medium	↑	FoxO1 regulated *Tfeb* by directly binding to its promoter	[49]
STAT1	Under normal conditions	↑	JAK2 induces translocation of STAT1 to the nucleus, where STAT1 binds to *Tfeb* promoter	[50]
KLF2	LSS	↑	By directly binding to *Tfeb* promoter	[51]
sXBP1	Mice with DIO	↑	By directly binding to *Tfeb* promoter	[52]
Noncoding RNAs				
MiR‐342‐3p	H_2_O_2_	↓	MiR‐342‐3p biding TFEB 3′ UTR and inhibiting *Tfeb* transcription	[56]
MiR‐128	Monocytes and lymphocytes from patients with AD; DA neurons	↓	Not mentioned	[36, 57]
Other regulators				
SMAD3	Diabetic nephropathy	↓	SMAD3 directly binds to the 3′‐UTR of *Tfeb* and inhibits its transcription	[54]
HDAC2	HDAC inhibitor SAHA	↓	By enhancing the transcription factor c‐MYC binding to *Tfeb* promoters and repressing its expression	[55]

*Note*: Differentiation medium, DMEM supplemented with 10% FBS, P/S, IBMX (0.5 mM), dexamethasone (1 μM), insulin (1 μg/ml), and rosiglitazone (2 μM).

Abbreviations: Ad‐sh‐RagA/B, adenovirus‐sh‐Rag proteins A and B; DA neurons, dopamine neurons; DIO, diet‐induced obesity; LSS, laminar shear stress; SAHA, suberoylanilide hydroxamic acid.

^a^ ↑, increase or activate; ↓, decrease or inhibit.

The positive feedback of TFEB on its own expression is also significantly enhanced during starvation.^30^ Transcription factor cellular ets‐2 gene (C‐ETS2) has been identified as another transcriptional activator of TFEB, which mediates TFEB expression and further mediates upregulation of lysosomal genes under oxidative stress.^48^ Forkhead box O1 (FoxO1), a transcription factor that controls mitochondrial function and morphology, interacts with *Tfeb* by directly binding its promoter, thereby regulating autophagy and mitochondrial uncoupling proteins (UCPs) expression in adipocytes.^49^ Studies involving ChIP assays have revealed that signal transducer and activator of transcription 1 (STAT1), a downstream mediator of the nonreceptor kinase Janus kinase 2 (JAK2) signaling pathway, binds to the *Tfeb* promoter, which in turn regulates the TFEB promoter activity, expression, and nuclear localization.^50^ Krüppel‐like factor 2 (KLF2), a shear stress‐responsive factor, upregulates *Tfeb* expression and promoter activity to exert anti‐inflammatory effects in endothelial cells.^51^ Defective autophagy and endoplasmic reticulum (ER) stress contribute to a variety of diseases. Spliced X‐box binding protein 1 (sXBP1), the key transcription factor that promotes adaptive unfolded protein response, occupies the −743 to −523 site of the *Tfeb* promoter and enhances TFEB transcription and autophagy in the context of obesity.^52^ Interestingly, a novel splicing variant of TFEB has recently been cloned, which comprises of 281 amino acids and lacks the HLH and Zip motifs present in the full‐length TFEB. One study found that the splicing variant of TFEB may act as a negative regulator of TFEB, and fine tune ALP activity during cellular stress.^53^ SMAD family member 3 (SMAD3) directly binds to the 3′‐UTR of *Tfeb* and inhibits its transcription, which triggers lysosome depletion in tubular epithelial cells in diabetic nephropathy.^54^ Finally, it has been reported that histone deacetylase 2 (HDAC2) enhance c‐MYC binding to *Tfeb* promoters to repress its expression.^55^

In addition to the regulation of promoter activity, recent studies have found that noncoding RNAs (ncRNAs) are involved in regulating TFEB transcription. A recent study showed that microRNA (miRNA)‐342‐3p directly targets TFEB by inhibiting H_2_O_2_‐induced autophagy, which contributes to exosome‐mediated heart repair.^56^ Different studies have predicted and validated TFEB as a target miR‐128‐mediated regulation of autophagy‐related gene transcription.^57^ In addition, some TFEB co‐activators, such as co‐activator‐associated arginine methyltransferase 1 (CARM1)^58^ and Yes‐associated protein (YAP),^59^ can also increase autophagy and lysosomal function by enhancing TFEB transcriptional activity.

### 3.2 Post‐transcriptional regulation of TFEB

**FIGURE 3:**
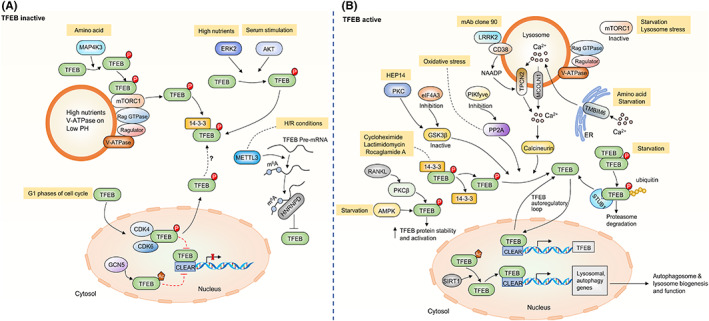
Post‐transcriptional regulation of TFEB. TFEB activity and nuclear translocation are regulated by its post‐transcriptional modification status. (A) Upon inactivation (under normal conditions), TFEB is phosphorylated by multiple kinases, and phosphorylated TFEB interacts with 14‐3‐3 proteins, remaining sequestered in the cytosol. Moreover, TFEB is acetylated by the histone acetyltransferase in nucleus, suppressing its transcriptional activity. (B) Upon activation (under conditions of lysosomal stress or starvation or mTOR inhibition), TFEB is dephosphorylated, unmasking its nuclear localization signal and driving transcription of itself and other CLEAR network of target genes. In addition, TFEB is phosphorylated by multiple kinases increasing its protein stability and activation. TFEB is deacetylated by the histone deacetylase, enhancing transcriptional levels of TFEB.

Under normal conditions, TFEB accumulates in the cytoplasm and is inactive. However, following conditions of stress, such as starvation or lysosomal dysfunction, TFEB rapidly translocates to the nucleus where it promotes transcription of its multiple target genes.^11^, ^26^ The phosphorylation and dephosphorylation of TFEB and its cytoplasmic‐nucleus shuttling are regulated by multiple pathways (Figures 2B and 3). TFEB activity and nuclear translocation are mainly controlled by its phosphorylation status.^11^

Mechanistic target of rapamycin complex 1 (mTORC1) phosphorylates serine residues in TFEB and plays a major role in the regulation of TFEB subcellular localization. Amino acids and the vacuolar H(+)‐adenosine triphosphatase ATPase (V‐ATPase) interact to active Rag GTPases, which then activate mTORC1 by recruiting it to the lysosomal membrane.^60^, ^61^ Interestingly, the active Rag GTPases also directly interact with TFEB to promote recruitment of TFEB to lysosomes.^62^ Under nutrient‐rich conditions or conditions in which lysosomal activity is adequate, mTORC1 binds to TFEB on the lysosomal surface, facilitating its phosphorylation on Ser142 and Ser211 respectively, resulting in cytoplasmic retention.^63^, ^64^ Mutations of either Ser142 or Ser211 into alanine (S142A, S211A, respectively) promote nuclear accumulation, similar to cells treated with the mTORC1 inhibitor Torin 1. Phosphorylation of Ser211 of TFEB promotes 14‐3‐3 proteins binding with its nuclear localization signal (NLS), thereby sequestering it in the cytoplasm.^63^, ^64^ In vitro kinase assays have shown that Ser122 is directly phosphorylated by mTORC1.^65^ However, the S122A mutation does not affect TFEB nuclear localization by itself, but enhances the effects of the S211A mutation. Conversely, a S122D mutation is sufficient to block the effects of the S211A mutation on TFEB nuclear translocation. Dephosphorylation at Ser122 is thought to be necessary for TFEB nuclear localization following mTORC1 inhibition.^65^ The mechanisms by which Ser142 and Ser122 phosphorylation affect TFEB subcellular localization are still unclear.

In addition to mTORC1, other kinases are known to phosphorylate TFEB and modulate its localization and activity. Phosphorylation of TFEB Ser142 by the extracellular signal‐regulated kinase 2 (ERK2) inhibits its nuclear localization and activity. Moreover, treatment with ERK inhibitors results in TFEB nuclear translocation.^11^ However, the relationship between mTORC1‐mediated and ERK2‐mediated phosphorylation of TFEB Ser142 is still unclear. In osteoclasts, protein kinase C β (PKCβ) phosphorylates multiple serine residues (i.e., Ser461, Ser462, Ser466, and Ser468) located in the last 15 amino acids of TFEB, which is important for TFEB protein stability and activation but does not affect its subcellular localization.^66^ TFEB can be phosphorylated by glycogen synthase kinase (GSK) 3β at Ser134 and Ser138 residues leading to its cytoplasmic retention. In contrast, GSK3β is inactivated by activated PKC and results in reduced phosphorylation and nuclear translocation of TFEB.^67^ One hypothesis is that the effects of GSK3β on TFEB may not be truly independent from mTORC1. Interestingly, TFEB Ser138 phosphorylation by GSK3β is primed by its Ser142 phosphorylation, and phosphorylation of both sites but not either alone, activates a previously unrecognized nuclear export signal (NES) of TFEB. Furthermore, during glucose limitation, GSK3β is inactivated by AKT in response to mTORC2 signaling and inhibits TFEB nuclear export.^68^ A recent study showed that TFEB is phosphorylated by AKT at residue Ser467 contributing to its cytosolic retention. TFEB S467A mutant shows an increased nuclear localization in normally fed conditions.^69^ When amino acids are plentiful, TFEB is phosphorylated by mitogen‐activated protein kinase 3 (MAP4K3) at residue Ser3. This phosphorylation modification is required for TFEB interaction with the mTORC1‐RagGTPase‐Ragulator complex and TFEB cytoplasmic retention, which also precedes for mTORC1 phosphorylation at Ser211 of TFEB.^70^ Another recent study showed that cyclin‐dependent kinases4/6 (CDK4/6) interacts with and phosphorylates TFEB at residues Ser142 in the nucleus in Torin‐1‐treated cells, thereby inactivating them by promoting their shuttling to the cytoplasm.^71^ Recently, a new finding showed that AMP‐activated protein kinase (AMPK) phosphorylates TFEB on highly conserved serine cluster 466, 467, and 469, leading to TFEB transcriptional activity upon nutrient starvation, folliculin (FLCN) depletion, or treatment with an inhibitor of mTORC1 or AMPK. However, S466A/S467A/S469A mutant blocks mTORC1‐regulated TFEB activity. These results suggest that TFEB transcriptional activity is dictated by an AMPK‐dependent mechanism, whereas nuclear translocation is dependent on mTORC1.^72^ Moreover, nonreceptor tyrosine kinase Abelson (c‐Abl) is a tyrosine kinase involved in cell signaling under physiological and pathophysiological conditions, which can also phosphorylate TFEB on tyrosine residue 75 (Y75) and Y173. Interestingly, TFEB Y173 is relevant for its retention in the cytoplasm.^73^ Inhibition of c‐Abl kinase activity in Niemann‐Pick type C (NPC) models, an autosomal recessive lysosomal storage disease (LSD) characterized by an accumulation of tissue cholesterol, TFEB translocases into the nucleus promoting the expression of its target genes in an mTOR‐independent manner, thereby inducing cholesterol‐lowering effects.^73^

As discussed above, the dephosphorylation of some key residues of TFEB is important for its activity. During starvation or conditions of increased reactive oxygen species (ROS), Ca^2+^ is released from the lysosome through the lysosomal calcium channel mucolipin 1 (MCOLN1), also known as the transient receptor potential mucolipin 1 (TRPML1), thereby increasing Ca^2+^ concentrations near the lysosome, which may lead to activation of the phosphatase calcineurin. Consequently, active calcineurin dephosphorylated TFEB, resulting in TFEB nuclear translocation and the subsequent transcription of target genes.^74^, ^75^ Interestingly, TRPML1 is also a direct transcriptional target of TFEB, suggesting the possibility of a positive feedback loop that involves TFEB and TRPML1.^28^ Recently, a study in immune cells showed that CD38 and leucine rich repeat kinase 2 (LRRK2) form a complex on the plasma membrane of B‐lymphocytes and macrophages. This study also showed that ligation of CD38 with the monoclonal antibody clone 90 results in the internalization of the complex and subsequent targeting to the endolysosomal system. LRRK2 kinase activation can promote activation of lysosomal nicotinic acid adenine dinucleotide phosphate (NAADP)‐Ca^2+^ signaling mediated by CD38, which results in the activation of protein phosphatase 3 (PPP3)/calcineurin, thereby dephosphorylating TFEB at Ser211 and prompting its nuclear translocation. Notably, the pathogenic LRRK2^G2019S^ mutant promotes TFEB activation via NAADP‐ TPCN2‐dependent calcium signaling and stabilizes TFEB by facilitating its C‐terminal phosphorylation.^76^ Recently, a new study showed that calcium release from the endoplasmic reticulum (ER) regulates autophagy function and lysosomal biosynthesis by regulating the TFEB nuclear translocation. Transmembrane BAX inhibitor motif containing 6 (TMBIM6), a component of the intracellular membranes of ER, is suggested to be a Ca^2+^ channel‐like protein.^77^, ^78^ TMBIM6 is present at the ER‐lysosome contact sites and play an important role in transferring Ca^2+^ to the lysosome. Under conditions of nutrient starvation or mTOR inhibition, TMBIM6 regulates Ca^2+^ release through MCOLN1/TRPML1 lysosomal channels, thereby activating calcineurin, which triggers TFEB nuclear translocation, autophagy induction, and lysosomal biogenesis.^79^ These results suggest that TFEB nuclear translocation may be affected by changes in intracellular calcium levels. In addition, protein phosphatase 2A (PP2A), which can be activated by oxidative stress, dephosphorylates TFEB at Ser109, Ser114, Ser122, and Ser211 residues, thereby facilitating TFEB activation. This suggests that TFEB may be involved in cellular responses to oxidative stress in an mTORC1‐independent manner.^80^ Moreover, a recent study also found that TFEB activation is dependent on PP2A rather than calcineurin/PPP3 Ser211 dephosphorylation during inhibition of phosphoinositide 5‐kinase (PIKfyve).^81^ Eukaryotic translation initiation factor 4A‐3 (eIF4A3) is an Asp‐Glu‐Ala‐Asp (DEAD) box‐family adenosine triphosphate (ATP)‐dependent RNA helicase, which is implicated in various aspects of RNA processing and function.^82^ Recently, Sakellariou et al. demonstrated that, upon loss of eIF4A3, TFEB is dephosphorylated and shuttles from the cytoplasm to the nucleus, due to the mis‐splicing of its upstream kinase, GSK3β^83^, ^84^ Furthermore, many translation inhibitors, including cycloheximide, lactimidomycin, and rocaglamide A, can facilitate the nuclear translocation of TFEB via dephosphorylation and 14‐3‐3 dissociation, thereby increasing autophagosome biogenesis.^85^ This result highlights the function of translation inhibitors as activators of TFEB, suggesting a novel biological role for translation inhibition in autophagy regulation.

Notably, growing evidence showed that the curcumin analogue, C1, a novel specific TFEB activator, promotes TFEB nuclear translocation in a phosphorylation‐independent manner.^35^, ^86^, ^87^ C1 directly binds to and activates TFEB, which promotes TFEB nuclear translocation by altering its structural conformation to expose nuclear localization signals.^86^ Interestingly, together with our collaborators, we have revealed that proteasome impairment facilitates TFEB dephosphorylation and nuclear translocation, which significantly increases the expression of a number of downstream TFEB target genes.^88^

In addition to phosphorylation and dephosphorylation modifications, other post‐transcriptional modifications are also involved in the regulation of TFEB function (Figure 3). Growing evidence has shown that acetylation and deacetylation modification of TFEB affects its activity and localization. TFEB K274 and K279 residues are acetylated by the histone acetyltransferase ‘general control non‐repressed protein 5’ (GCN5) in the nucleus, which suppresses its transcriptional activity by disrupting its dimerization and the binding to its target gene promoters.^89^ In cancer cells, suberoylanilide hydroxamic acid (SAHA), a well‐established HDAC inhibitor, promotes acetylation of TFEB at K91, K103, K116, and K430 by enhancing the interaction of acetyl‐coenzyme A acetyltransferase 1 (ACAT1) with TFEB. This activates TFEB transcriptional activity and promotes its nuclear translocation.^90^ In contrast, inhibition of the cytosolic deacetylase, HDAC6, enhances TFEB acetylation, which in turn increases TFEB nuclear localization in experimental kidney disease, but the deacetylation site on TFEB and the exact mechanisms behind this are still unclear.^91^ Sirtuin 1 (SIRT1), a nicotinamide adenine dinucleotide (NAD^+^)‐dependent histone deacetylase, directly interacts with and deacetylated TFEB at K116 residue in the nucleus of the microglia treated with fibrillar Aβ (fAβ), thereby enhancing transcriptional levels of TFEB. Furthermore, overexpression of the deacetylated TFEB at K116R mutant significantly increases the lysosomal function.^92^ However, further studies are required to elucidate the functionality of TFEB acetylation in controlling its transcriptional activity.

N^6^‐methyladenosine (m^6^A) modification is the most prevalent modification in eukaryotic mRNAs.^93^ Recent studies have found that the m^6^A modification is strongly linked to increased mRNA degradation.^94^ Previous studies have suggested that methyltransferase like 3 (METTL3) binds to the 3’‐UTR end of TFEB to methylate two m^6^A residues, which promote the interaction of the RNA binding protein heterogeneous nuclear ribonucleoprotein D0 (HNRNPD) with TFEB pre‐mRNA, subsequently decreasing the transcriptional activity of TFEB. In turn, TFEB downregulates the mRNA stability of METTL3, thereby establishing a negative feedback loop.^95^

Ubiquitination also plays an important role in TFEB activation. One study showed that phosphorylation of Ser142 and Ser211 mediates TFEB‐targeted degradation by the ubiquitin‐proteasome system via binding to the E3 ubiquitin ligase, STIP1 homology and U‐Box containing protein 1 (STUB1). This suggests that the activity and stability of TFEB in cells may be jointly regulated by phosphorylation and ubiquitination.^96^ SUMOylation does not promote protein degradation like ubiquitination, but rather strengthens protein stability and affects protein transcription activity. It has been reported that TFEB contains the sumoylation consensus sequence ΨKXE, with SUMOylation of TFEB at a lysine site leading to its decreased transcriptional activity in vivo.^97^

## 4 IMPAIRED TFEB SIGNALING AS A CONTRIBUTOR TO THE PATHOGENESIS OF NEURODEGENERATIVE DISEASES

**FIGURE 4:**
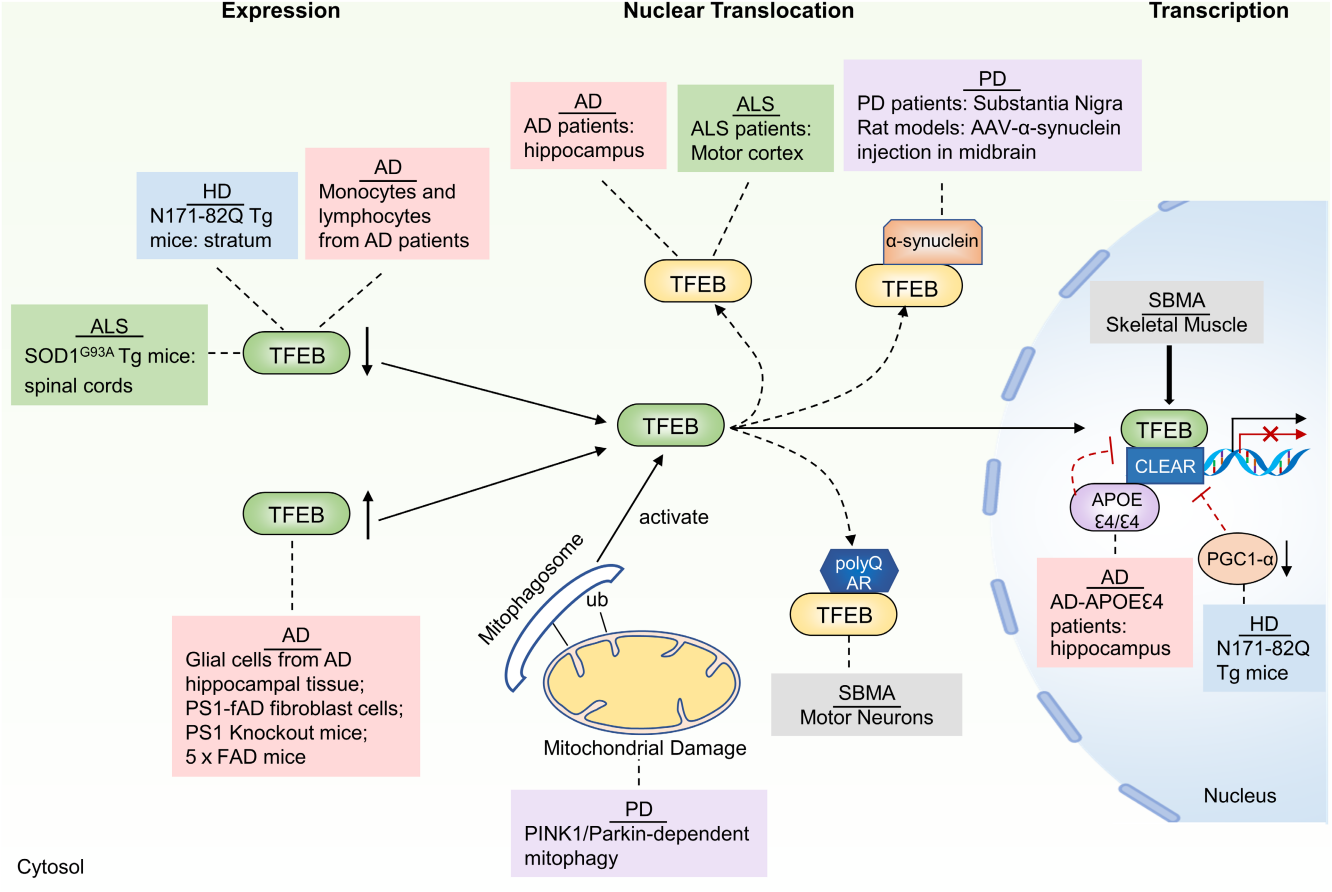
TFEB dysregulation in neurodegenerative disease. TFEB expression, nuclear translocation, and its transcription activity are dysregulated in neurodegenerative disease. TFEB expression reduces in monocytes and lymphocytes of AD, HD, ALS, and increases in glial cells of AD and PS1 knockout mice. TFEB nuclear translocation reduces in AD, PD, ALS, and motor neurons of SBMA. The transcription activity of TFEB is dysfunctional in AD, HD, and skeletal muscle of SBMA.

Intracellular accumulation of aberrant protein inclusions is the primary molecular pathogenic event of neurodegenerative diseases. Some protein aggregates associated with neurodegenerative diseases reportedly interact with TFEB. Indeed, studies of postmortem tissues from patients and animal models of neurodegenerative diseases have suggested that impaired TFEB signaling, including abnormal TFEB expression, changes in TFEB subcellular localization, and that abnormal expression of TFEB‐targeting ALP genes may contribute to the pathogenesis of neurodegenerative diseases (Figure 4).

### 4.1 Alzheimer's disease

AD is the most common neurodegenerative diseases in the elderly and is clinically characterized by progressive cognitive impairment, often accompanied with psychobehavioural disturbances and language impairment in later stages. Extracellular amyloid beta (Aβ) plaques, intracellular neurofibrillary tangles (NFTs), and extensive neuronal degeneration are the three major pathological hallmarks of AD.^98^ The production of Aβ, which is considered a crucial process in AD pathogenesis, is the proteolysis product of amyloid‐β precursor protein (APP), which is overexpressed in AD.^99^ Aβ is generated through a two‐step processing of APP: cleavage of APP by β‐site APP cleaving enzyme 1 (BACE1) to generate a β‐C‐terminal fragment (β‐CTF), which upon cleavage by γ‐secretase produces Aβ. In addition, cleavage of APP by α‐secretases within the Aβ domain prevents Aβ production.^100^, ^101^ Moreover, hyperphosphorylated Tau protein is the main component of NFTs.^102^ Multiple lines of evidence support that the ALP contributes to the removal of abnormally aggregated Aβ and tau proteins.^103^, ^104^, ^105^ As a major regulator for ALP, TFEB plays an important role in the pathogenesis of AD. However, the role of TFEB currently showed in AD pathogenesis is controversial. An analysis in monocytes and lymphocytes from AD patients revealed a significant decrease in the expression of TFEB and its target lysosomal genes, suggesting its possible role in lysosomal deficits in AD.^57^ A separate study later confirmed that the nuclear TFEB was striking reduced in brain tissue samples from AD patients. Furthermore, its reduction is positively correlated with AD pathology.^106^ Similarly, TFEB nuclear transport was significantly impeded in an in vitro model of double presenilin knockout cells, indicating that TFEB cytosolic retention may contribute to AD pathogenesis.^107^ Another study showed that *Tfeb* mRNA expression in microdissected CA1 neurons were unchanged in AD, whereas expression levels were significantly increased in AD hippocampal tissue. Notably, the study also demonstrated that TFEB nuclear translocation significantly increased in glial cells of AD hippocampal tissue, indicating an important role for TFEB in the glia of CA1 hippocampus.^108^ An in vitro study using primary microglia showed that Aβ_1‐42_ induced reduction of the nuclear TFEB in a dose‐dependent manner and significantly inhibited the expression of osteoporosis‐associated transmembrane protein 1 (OSTM1), a vital molecule involved in lysosome acidification, resulting in lysosomal dysfunction.^109^ In contrast, there was a significant increase in TFEB mRNA in AD patient‐derived fibroblasts carrying the familial presenilin‐1 (PS1) A246E mutation.^110^ Another study of presenilin‐1 conditional knock‐out mice further revealed significant upregulation of a subset of CLEAR network genes related to lysosomal biogenesis, although failing to reveal a statistically significant difference in *Tfeb* mRNA expression.^111^ Similarly, upregulation of TFEB target genes was also reported in 5xFAD mice, which contains five familial mutations related to AD.^112^ In addition, computational modeling and DNA pull‐down binding assays showed that APOE ɛ4, a key genetic risk factor for sporadic AD, may compete with TFEB for binding to CLEAR elements in the promoters of important genes involved in the ALP, including SQSTM1, MAP1LC3B, and LAMP2, thereby suggesting inhibition of the CLEAR network in APOE ɛ4/ɛ4 AD patients.^113^ In agreement with this hypothesis, neuron‐specific knockout of TFEB in the hippocampus of 2‐month‐old mice significantly increased the accumulation of total Aβ and paired helical filament (PHF) pTau in the brain, further suggesting that TFEB plays a key role in AD neuropathology.^107^ Taken together, these studies clearly demonstrate the role of abnormal TFEB expression and activity, and subsequently impaired ALP, in the pathogenesis of AD. However, upstream events affecting TFEB expression and nuclear translocation in AD remain unclear.

### 4.2 Parkinson's disease

PD is the second‐most prevalent neurodegenerative disorder affecting dopaminergic neurons in the substantia nigra pars compacta (SNpc). The main pathological hallmarks of PD are the accumulation of proteinaceous cytoplasmic inclusions (Lewy bodies) composed of misfolded and aggregated α‐synuclein in SNpc dopaminergic neurons and the progressive loss of dopaminergic neurons.^114^ In an early study, autophagic degeneration are observed in the dopaminergic neurons of the substantia nigra of PD patients.^115^ Studies indicate that α‐synuclein hinders autophagy by blocking ER‐Golgi vesicular trafficking, while overexpression of Rab1 linked to macroautophagy regulation can rescue α‐synuclein‐induced autophagy impairment.^116^ Furthermore, accumulation of autophagic vesicles and lysosomal deficiency were observed in other in vitro and rodent models of PD, as well as in postmortem PD samples.^36^, ^37^ Moreover, nuclear expression of TFEB is significantly reduced in postmortem human PD midbrains. Furthermore, α‐synuclein and 14‐3‐3 were found to bind to TFEB, sequestering TFEB into the cytoplasm and preventing its activation in the adeno‐associated virus (AAV)‐α‐synuclein rat model. Interestingly, TFEB colocalizes with α‐synuclein and has also been observed in Lewy body‐containing nigral neurons in human PD brains.^36^

Mitophagy refers to the selective degradation of damaged mitochondria by autophagy. The kinase PTEN‐induced kinase 1 (PINK1) and ubiquitin ligase Parkin can regulate the biological process of mitophagy.^117^ Importantly, mutations in PINK1 and Parkin are closely associated with early‐onset recessive PD.^118^, ^119^ As reviewed by Pickrell et al.,^120^ accumulation of impaired mitochondria in PD is caused by defective mitophagy. The roles of TFEB in mitophagy have been reported in few studies.^29^, ^121^ During mitophagy, TFEB translocases into the nucleus and activates transcriptional activity in a PINK1‐ and Parkin‐dependent manner, which requires the activation of Atg9A and Atg5.^29^ TFEB translocates to the nucleus following mitophagy induction to increase its expression as well as lysosomal proteins expression. In mice with a Parkin^Q311X^ mutation, the mutant Parkin fails to binds and ubiquitinates the parkin‐interacting substrate (PARIS), a transcriptional repressor of PGC1‐α, in turn leading to a decrease of PGC1α‐TFEB signaling, which plays a crucial role in the clearance of damaged mitochondria and mitochondrial biogenesis.^122^ Furthermore, increased expression of TFEB in cells significantly increase the expression level of PGC‐1α mRNA, resulting in increased mitochondrial content.^121^ These findings revealed a role for TFEB in mitochondrial quality control, but the specific molecular mechanism in PD remains unclear.

### 4.3 PolyQ diseases

HD is a common polyQ neurodegenerative disease caused by repeat expansion of CAG trinucleotide in the first exon of the huntingtin (Htt) gene.^123^ Increased numbers of autophagic vacuoles have been observed in many HD mouse models and in non‐neuronal cells from patients with HD.^124^ Furthermore, the cargo‐adaptor p62 and LC3‐II are specifically increased in the striatum of HD transgenic mice.^125^ However, work by the Cuervo group found expansion of autophagy compartments was never accompanied by the expected increase in autophagy‐mediated degradation in HD cells. Instead, they found reduced amounts of cytosolic cargo inside ‘empty’ autophagosomes caused by aberrant p62‐polyQ‐Htt interaction, which in turn yields a low protein degradation rate in HD samples.^124^ Interestingly, mas Htt has been proposed to act as an autophagy adaptor protein, serving as a scaffold for the ULK1 and Atg1 autophagy initiation complexes.^126^, ^127^ Thus, various lines of investigation implicate dysfunctional autophagy as part of HD pathology.

TFEB expression and function was first shown to be reduced in the striatum of HD N171‐82Q transgenic mice.^40^ PPARGC1A/PGC‐1α, a regulator of mitochondrial biogenesis and oxidative stress, is a downstream target gene of TFEB in the regulation of lipid catabolism.^30^ Interestingly, TFEB was identified as a downstream mediator and transcriptional target of PGC‐1α in the striatum of HD mice. Moreover, PGC‐1α overexpression enhances Htt turnover and eliminates protein aggregation by promoting TFEB transcription and activation. Furthermore, defects in TFEB and its target genes are corrected by overexpression of PGC‐1α.^40^ Changes in lipid/sterol/lipoprotein pathways were observed in a genome‐wide expression analysis of mouse embryonic stem cells expressing endogenous Htt with increasing lengths of polyglutamine, suggesting a possible link between mutant Htt and lipid metabolism.^128^ The dual feedback loop of bidirectional activation between TFEB and PGC1α may play an important role in regulating the relationship between these two biological processes in HD. However, further studies are required to confirm this phenomenon.

Spinal and bulbar muscular atrophy (SBMA) is a neuromuscular disorder caused by expansion of a CAG repeats in the first exon of the androgen receptor (AR) gene. The main pathological hallmark of SBMA is adult‐onset proximal muscle weakness due to lower motor neuron degeneration in the spinal cord and brain stem.^129^ An elegant study by Cortes et al.^130^ showed that polyQ‐AR impaires autophagic flux by interfering with TFEB transactivation in motor neuron‐like cells. Furthermore, overexpression of TFEB rescues the metabolic and autophagic flux defects caused by polyQ‐AR.

Interestingly, the TFEB activity is reduced in SBMA motor neurons and patient‐derived neuronal progenitor cells (NPCs), while analysis of quadriceps muscle samples from SBMA YAC AR100 mice yields evidence of a dramatic upregulation of TFEB target genes.^130^ Similarly, in another in vivo and in vitro study of SBMA, polyQ‐AR was found to promote autophagy by activating TFEB activity.^131^ These studies suggest a muscle‐specific process of supraphysiological induction of TFEB in diseased SBMA muscle cells. Although the mechanism by which polyQ‐AR contributes to different aspects of TFEB dysregulation in different tissues remains unknown, it is worth noting that systemic administration of autophagy therapies may have deleterious effects on SBMA.

### 4.4 Amyotrophic lateral sclerosis

ALS is the most common adult‐onset motor neuron disorder characterized by selective loss of motor neurons in the motor cortex, brainstem and spinal cord. Familial ALS cases are caused by mutations in a number of genes, including superoxide dismutase 1 (SOD1), chromosome 9 open reading framework 72 (C9orf72), TAR DNA binding protein (TDP‐43), and valosin‐containing protein (VCP).^132^, ^133^ In ALS, postmortem patient tissue studies have revealed evidence of autophagy dysregulation in motor neurons.^132^ In addition, autophagosome accumulation was observed in degenerating motor neurons of ALS patients and was often located adjacent to p62‐positive inclusions.^134^ Abnormal accumulation of mutant SOD1 protein aggregates is a key neuropathological hallmark responsible for the progressive loss of motor neurons in ALS.^135^ Stage‐dependent alterations in TFEB expression has been reported in SOD1^G39A^ transgenic mice.^136^ In this study, TFEB was upregulated in the early stage of disease, but then reduced in the spinal cord at the middle and end stages of the disease. In vitro TFEB overexpression has been shown to increase cell survival and proliferation by inducing autophagy, although the effect of autophagy on SOD1‐aggregation clearance has not been studied.^136^ Furthermore, studies suggest that nuclear TFEB levels were reduced in ALS patient brain samples, suggesting decreased TFEB activity in ALS.^106^ TFEB expression and nuclear translocation have been shown to increase in a C9orf72 knock‐out mouse model.^137^ A recent study in *C. elegans* found that deletion of C9orf72/ALFA‐1 leads to nuclear translocation of HLH‐30/TFEB, subsequently leading to activation of lipolysis and premature lethality. Furthermore, decreased TFEB phosphorylation and increased nuclear translocation of TFEB were observed in C9orf72‐deficient cells under conditions of amino acid stimulation, which were rescued by overexpression of the active forms of Rag GTPases. This suggests that C9orf72 regulates TFEB activity via Rag.^138^ TDP‐43, another ALS‐associated gene, has been identified as a negative regulator of autophagy and TFEB activity.^139^ Deletion of TDP‐43 significantly increases nuclear translocation of TFEB by targeting raptor, an adaptor of mTORC1, and enhances the expression of ALP‐related genes. Furthermore, loss of TDP‐43 also hindered the fusion of autophagosomes with lysosomes by downregulating dynactin1, resulting in an accumulation of immature autophagic vesicles and further impairment of ALP function.^139^ Mutations in the VCP gene were recently reported to be the cause of familial ALS. TFEB is activated in VCP‐knockout muscle, and the activation and nuclear localization of TFEB persists upon VCP inactivation or mutation.^140^ Thus, while the study of TFEB dysregulation in motor neuron disease is a very interesting topic, the exact role of autophagy in the pathogenesis of ALS remains unclear. Future studies are required to identify the specific mechanisms of autophagy dysfunction in ALS, and to determine whether TFEB or autophagy itself may be a potentially viable therapeutic target.

## 5 TFEB AS A THERAPEUTIC TARGET FOR NEURODEGENERATIVE DISEASES

Increasing evidence has shown that the accumulation of protein aggregates and ALP dysfunction are the major pathogenic mechanisms for neurodegenerative diseases.^141^ As a master regulator of ALP pathways, TFEB has become an attractive target for alleviating ALP dysfunction in neurodegenerative diseases.

### 5.1 Therapeutic effects of TFEB overexpression in neurodegenerative diseases

The beneficial effects mediated by TFEB have been demonstrated in multiple mouse and cell models of AD addressing Aβ and tau pathology.^33^, ^142^, ^143^ With respect to Aβ, TFEB overexpression rescues the autophagic flux, accelerates Aβ_1–42_ degradation by regulating the autophagy‐lysosome pathway, and alleviates Aβ_1–42_‐induced toxicity by reducing oxidative stress.^144^ Similarly, intracranial stereotaxic injection of AAV‐TFEB in hippocampal neurons of APP/PS1 mice leads to a reduction of APP, Aβ production, and amyloid plaque load by accelerating flux of the endosome‐lysosome pathway.^142^ Interestingly, nonneuronal cells in AD may also benefit from TFEB induction. In microglia, deacetylation of TFEB by SIRT1 stimulates fibrillar Aβ (fAβ) degradation by facilitating lysosomal biogenesis and reduces amyloid plaques in the brains of APP/PS1 mice.^92^ In APP transgenic mice specifically overexpressing TFEB in hippocampal astrocytes, TFEB mainly localizes in the nuclei of astrocytes and enhances lysosome function, resulting in reduced Aβ levels and amyloid plaque load. These data suggest that TFEB can also facilitate Aβ clearance in astrocytes.^34^ A recent study indicates that exogenous TFEB in both cells and the 3xTgAD mouse model strongly reduces C99 load by increasing the expression of cathepsins, key proteases involved in C99 degradation. This suggests that TFEB activation is an important strategy for preventing the accumulation of the early neurotoxic catabolite of AD.^143^ A disintegrin and metalloproteinase 10 (ADAM10) has recently emerged as the major α‐secretase responsible for APP processing,^145^ which plays a crucial role in axon guidance and spine density regulation.^146^, ^147^ There is now definitive evidence that reduction of ADAM10 activity and impaired trafficking to the synapse of ADAM10 can cause AD, thereby suggesting that inadequate ADAM10 activity is likely the cause of AD.^148^, ^149^ A recent study showed that TFEB overexpression increased the expression of mature ADAM10 and its enzyme activity in both the cortex and hippocampus of flag‐TFEB mice, resulting in increased generation of soluble‐APP‐α (sAPPα). Furthermore, the TFEB‐induced increase in mature ADAM10 protein levels is mediated via PPAR‐α.^150^ For tau pathology, TFEB overexpression in the brain of a rTg4510 mouse model of tauopathy dramatically reduces tau pathology, synaptic deficits, neurodegeneration and animal behavioral deficits. The proposed mechanism of this effect may be that TFEB induces ALP through upregulation of phosphatase and tensin homolog (PTEN), which is a lipid phosphatase that antagonizes PI3K‐Akt‐mTOR signaling.^33^ In agreement with this conclusion, TFEB overexpression enhances uptake of extracellular tau protein, promotes lysosomal activity in primary astrocytes, reduces the pathology induced by hyperphosphorylated and misfolded tau protein, and significantly attenuates tau spreading in astrocytes in PS19 tauopathy mice.^151^ Similarly, neuron‐specific TFEB overexpression significantly reduces the expression of toxic p‐tau and the number of lipofuscin puncta in the cortex and hippocampus of P301S tauopathy mice, and attenuates the learning and memory deficits in mice.^152^ In a recent study, TFEB was found to regulate the secretion of truncated mutant tau lacking a microtubule‐binding repeat (MTBR) by promoting lysosomal exocytosis. This study also showed an that this process is dependent on the lysosomal calcium channel, TRPML1.^153^ Thus, the beneficial effects of exogenous TFEB expression on the clearance of Aβ and tau in in vitro and in vivo models of AD are clear, while the contribution of endogenous TFEB to AD progression is less well defined.

So far, various potential treatments for PD have been developed in PD cell and animal‐based models, but none of them have achieved satisfactory efficacy or have more or less side effects.^154^, ^155^ Accumulating evidence suggests that TFEB may be a possible therapeutic target for PD. In PD model, TFEB overexpression afforded robust neuroprotection via the clearance of α‐synuclein aggregation.^36^, ^156^ TFEB overexpression counteracts atrophy and preserves neuronal integrity and function in the MPTP mouse model. Notably, TFEB overexpression in wild type mice induces a neurotrophic effect that involves cell growth and higher amounts of releasable dopamine. Additionally, TFEB overexpression can also activate mitogen‐activated protein kinases (MAPK) 1/3 and AKT pro‐survival pathways and increase phosphorylation of mTORC1 effectors 4E‐BP1 and S6K1, promoting protein synthesis, all of which are the biological processes that are unrelated to ALP.^157^ Notably, TFEB overexpression has no effect on the expression and activity of mTORC2. Collectively, these findings suggest that TFEB may be a very promising target to counteract neurodegeneration by improving autophagy dysfunction and other biological processes in PD.

Studies from different groups have reported that overexpression of TFEB can reduce mHtt in both cellular and animal models of HD.^26^, ^40^, ^158^ However, many recent studies have also shown that TFEB overexpression does not reduce mutant HTT aggregation in a mouse model of HD, possibly related to the formation of mHtt‐TFEB coaggregation mediated by a prion‐like domain (PrLD) near the N‐terminus of TFEB.^159^, ^160^ This result suggests that therapeutic strategies directly targeting TFEB to clear mHtt aggregates may have some limitations.

Some studies have reported that in vitro overexpression of TFEB increases cell survival and proliferation to improve ALS pathogenesis, making TFEB a promising target for the development of novel drug and gene therapeutics for ALS.^136^ However, the protective effect of TFEB overexpression on ALS requires further experimental study explored in animals.

### 5.2 Therapeutic effects of TFEB‐targeted agonists in neurodegenerative diseases

A growing body of evidence has shown that some small molecules and natural products derived from traditional Chinese medicine (TCM) have therapeutic promise for neurodegenerative diseases. These have been reported to activate TFEB through multiple mechanisms, including direct TFEB activation, mTORC1 inhibition, AKT inhibition, as well as Ca^2+^‐dependence, among others. The TFEB‐activating targets/pathways and the effects of these TCM‐derived natural compounds and small molecules in neurodegenerative diseases models are summarized in Table 2.

**TABLE 2:** TFEB‐targeted agonists and their protective effects in neurodegenerative disease

Compound	Mechanism of TFEB activation	Disease	Cell/animal models	Effects	References
Natural small‐molecule compounds					
Curcumin	Promote TFEB nuclear translocation via the phosphorylation of GSK‐3β at the serine 9 residue	AD, PD	SH‐SY5Y cells expressing mutant APP or mutant α‐synuclein	Degrade aggregated APP and α‐synuclein via the ALP pathway	[165]
Curcumin analog C1	Bind to TFEB at the N terminus and promote TFEB nuclear translocation without inhibiting MTOR activity	PD, AD	MPTP mouse model, 6‐OHDA/AA‐induced cell models, 5xFAD mice, P301S mice, 3xTg‐AD mice	Reduce APP, CTF‐β/α, β‐amyloid peptides and tau aggregates	[35, 38, 86, 166]
Curcumin analog E4	Reduce the level of p‐TFEB (Ser142) via inhibiting AKT‐mTORC1 pathway	PD	PC12 cells treated with MPP^+^	Promote α‐synuclein degradation	[167]
Trehalose	Promote TFEB nuclear translocation; Promote TFEB nuclear translocation through enhancing its dephosphorylation level at S142 site mediated by PPP3CB upregulation; Promote TFEB nuclear translocation and CLEAR network activation via diminishing AKT activity	PD, ALS, SBMA, Batten disease	M17 cells treated with MPP^+^, NSC34 cells treated with AR.Q46 or SOD1^A4V and G93A^, SH‐SY5Y cells overexpressing TDP‐43, patient‐derived JNCL fibroblasts, Cln3^Δex7–8^ mice	Attenuate MPP^+^‐induced cell death, enhance degradation of polyQ‐AR, TDP‐43 and SOD1, enhance clearance of proteolipid aggregates	[37, 69, 170, 171]
Genistein	Increase *Tfeb* mRNA expression and promote TFEB nuclear translocation	NPCD	NPC1 patient fibroblasts	Alleviate cholesterol accumulation	[176, 179]
Ouabain	Promote TFEB nuclear translocation through enhancing its dephosphorylation level	AD	SH‐SY5Y cells overexpressing TauP301S, drosophila tau model, TauP301L mice	Reduce phosphorylated tau, attenuate memory impairment	[185]
Hep14	Inactivate GSK3β by activating PKC, leading to reduced phosphorylation and increased nuclear translocation of TFEB	HD, AD	HepG2 cells treated with oleic acid, HeLa cells overexpressing 97Q, APP/PS1 mice	Facilitate clearance of polyQ‐Htt aggregates and lipid droplets, ameliorate amyloid β plaque formation in mouse brains	[67]
Gypenoside XVII	Release of TFEB from the TFEB/14‐3‐3 complex leads to TFEB nuclear translocation	AD	APP/PS1 mice	Facilitate clearance of APP, Aβ_40_, and Aβ_42_, attenuate the spatial learning and memory deficits	[186]
Cinnamic acid	Transcriptionally upregulate *Tfeb* via PPARα activation	AD	5xFAD mice	Reduce amyloid‐β plaque burden and improve memory deficit	[187]
Pseudoginsenoside F11	Promote TFEB nuclear translocation via mTOR inhibition	AD	Primary rat microglial cells treat with oligomeric Aβ	Increase the degradation of oligomeric Aβ in microglia	[190]
Fisetin	Promote TFEB nuclear translocation via mTOR inhibition	AD	T4 cells treated with doxycycline	Promote degradation of phosphorylated tau	[192]
Paeoniflorin	Increase the transactivation of TFEB, increase TFEB expression via the upregulation of NF‐YA, and promote TFEB nuclear translocation	SBMA	NSC34 cells overexpressing mutant (97Q) AR, AR‐97Q mice	Enhance both the UPS and autophagy systems, mitigate the behavioral and pathological impairments	[193]
Chlorogenic acid	Promote TFEB nuclear translocation via mTOR inhibition	AD	Aβ25‐35‐exposed SH‐SY5Y cells, APP/PS1 mice	Alleviate neuron damage and cognitive impairment	[194]
Qingyangshen	Increase expression of TFEB and PPARα	AD	HT‐22 cells overexpressing APP and Tau, 3xTg AD mice	Reduce expression of APP and phospho‐Tau, improve learning and spatial memory behavior	[198]
Celastrol	Induce dephosphorylation of TFEB (S142 and S211) via mTORC1 inhibition	AD	P301S Tau and 3xTg mice	Reduce phosphorylated Tau aggregates, attenuate cognitive deficits, enhance autophagy and lysosomal biogenesis	[199]
Small molecule inhibitor and clinical drugs					
Rapamycin and its analogs CCI‐779	Induce dephosphorylation of TFEB via mTORC1 inhibition; restore PGC1α‐TFEB signaling in a manner not requiring parkin activity	PD	AAV‐α‐synuclein injected rats, Parkin Q311X mutant mouse	Improve motor impairment, increase survival of dopamine neurons, reduce the toxic oligomeric α‐synuclein, abrogate mitochondrial impairment	[36, 122
Cyclodextrin	Enhance TFEB expression and prompt TFEB nuclear translocation	PD, NPCD	H4 cells overexpressing α‐synuclein, NPC1 patient‐derived fibroblasts	HPβCD enhance autophagic clearance of α‐synuclein aggregates, HPγCD rescue the cholesterol accumulation in lysosomes	[39, 206]
STI‐571	Promote TFEB nuclear translocation by abrogating c‐Abl‐induced phosphorylation of GSK3b‐Tyr216	PD	SN4741 cells and primary midbrain neurons treated with MPP^+^	Alleviate MPP^+^‐induced impairment of ALP function and MPP^+^‐induced cells death	[207]
Veliparib	Enhance TFEB nuclear transcription via SIRT1 mediated downregulation of mTORC1 and reduce nuclear export of TFEB by attenuating the TFEB‐CRM1 interaction	PD	α‐synuclein A53T animal model, SN4741 cells overexpressing α‐synucleinA53T	Prevent neurodegeneration and improve motor activity in PD mouse models	[208]
SB203580	Promote TFEB nuclear translocation by inhibiting p38‐induced phosphorylation of TFEB (S211)	PD	α‐synuclein A53T animal model	Improve the motor activity, alleviate abnormal accumulation of α‐synuclein	[209]
Celecoxib	Increase TFEB expression	PD	SH‐SY5Y cells treated with 6‐OHDA or paraquat (PQ)	Reduce 6‐OHDA‐and PQ‐induced dopaminergic cell death	[210]
Dynasore	Repress the lysosomal localization of mTOR and block the activity of mTORC1, which in turn enhance the nuclear translocation of TFEB	HD	HEK 293 cells overexpressing Nhtt60Q	Promote the clearance of protein aggregates formed by mutant polyQ‐Htt protein	[211]
Aspirin	Induce the activation of PPARα and stimulated the transcription of *Tfeb* via PPARα	AD	5xFAD mice, primary mouse astroglia	Decrease amyloid plaque pathology	[212, 213]
Ibudilast	Enhance TFEB nuclear translocation by inhibiting the mTORC1 activity	ALS	HEK 293 overexpressing TDP‐43 or SOD1^G93A^, NSC‐34 cells	Enhance the clearance of TDP‐43 and SOD1 protein aggregates	[214]
Others					
Acylated ghrelin	Upregulate TFEB expression and increase TFEB nuclear translocation	PD	SH‐SY5Y cells treated with 6‐OHDA, 6‐OHDA‐induced rat model	Ameliorate 6‐OHDA‐induced neurotoxicity and 6‐OHDA‐induced motor dysfunction	[216]
PNU282987	Induce TFEB nuclear translocation	ALS	N2a cells overexpressing SOD1^G85R^	Exhibit significant neuroprotective effects against SOD1^G85R^ induced neurotoxicity	[217]
F‐SLOH	Promote TFEB nuclear translocation via MAPK1/ERK2 inhibition and PP2A activation	AD	5xFAD, 3xTg‐AD mice, N2a overexpressing human APP^Swe/Ind^ or P301L‐Tau	Enhance the clearance of APP, Aβ oligomers, and hyperphosphorylated Tau aggregates	[219]

#### 5.2.1 Natural small‐molecule compounds

##### Curcumin and its analogs

Curcumin, a natural polyphenol extracted from turmeric (*Curcuma longa* L.), has been reported to possess multiple pharmacological properties.^161^ Curcumin has been shown to enhance autophagy by suppressing the PI3K‐Akt–mTOR pathway and activating the ERK1/2 pathway.^162^, ^163^, ^164^ Recently, a study found that curcumin promotes TFEB nuclear translocation by inhibiting GSK‐3β activity and degrades aggregated APP and α‐synuclein via the TFEB‐autophagy/lysosomal pathway in SH‐SY5Y cells.^165^

Due to its low bioavailability, several curcumin derivatives have been chemically synthesized to enhance its efficacy. A synthesized curcumin monocarbonyl derivative called C1(1,5‐bis[2‐methoxyphenyl]penta‐1,4‐dien‐3‐one) has been identified as a novel mTOR‐independent activator of TFEB.^86^ Compound C1 directly binds to the N terminus of TFEB and promotes it entry into the nucleus, without affecting TFEB phosphorylation or inhibiting upstreaming regulators of TFEB, including mTORC1 and MAPK1.^86^ Compound C1 enhances autophagy and lysosomal activity by activating TFEB, and reducing APP, APP C‐terminal fragments (CTF‐β/α), β‐amyloid peptides and tau aggregates in three AD animal models that represent β‐ APP pathology (5xFAD mice), tauopathy (P301S mice), and the APP/Tau combined pathology (3xTg‐AD mice). In addition, compound C1 improves the motor and cognitive function in animal models of AD.^35^ The protective effects of compound C1 have also been examined in PD cellular and animal models. In 6‐hydroxydopamine (6‐OHDA)/ascorbic acid (AA)‐induced models of PD, compound C1 has been shown to enhance TFEB nuclear translocation and autophagy to exert neuroprotective effects. Compound C1 significantly reduces oxidative stress‐induced dopaminergic cell death and improves motor impairment. Moreover, these effects are prevented by silencing of TFEB.^38^ Recently, the curcumin analog C1‐based nanoscavenger (NanoCA), a self‐assembled product from curcumin analog C1 molecules and polyethylene glycol, activates TFEB nuclear translocation in an mTOR‐independent manner, and eventually promotes the autophagic degradation of α‐synuclein. Furthermore, the brain‐targeted NanoCA also promotes clearance of α‐synuclein aggregates and improves the behavioral deficits of MPTP‐intoxicated PD animal model, suggesting a promising approach for PD intervention.^166^ Curcumin analog E4, another curcumin derivative, potently activates TFEB via AKT‐mTORC1 inhibition. Compound E4 enhances autophagy flux and lysosomal biogenesis, promotes α‐synuclein degradation, and protects against MPP^+^‐induced cytotoxicity in neuronal cells.^167^

##### Trehalose

Trehalose, a disaccharide homologous to sucrose, has recently been shown to have potential utility in neuroprotection by reducing the aggregation of misfolded proteins and promoting the clearance of abnormal protein aggregates by autophagy induction.^168^, ^169^ Trehalose enhances the degradation of polyQ‐AR (which is associated with SBMA), TDP‐43, and SOD1 (which are associated with ALS) via the TFEB pathway in an mTOR‐independent manner, while silencing TFEB disturbs the pro‐degradation activity of trehalose.^170^, ^171^ Similarly, trehalose is able to activate TFEB, as indicated by its nuclear translocation upon treatment, and attenuates MPP^+^‐induced cell death.^37^ In addition, trehalose also activates TFEB in an indirect manner. Trehalose reportedly induces TFEB nuclear translocation and upregulate TFEB target genes, in a manner that is mediated by PPP3CB downregulation.^170^ Moreover, a recent study revealed that trehalose can activate TFEB by diminishing AKT activity, an upstream kinase that phosphorylats TFEB at Ser467, thereby enhancing the clearance of proteolipid aggregates and reducing neuropathology in a mouse model of Batten disease, a prototypical neurodegenerative disease presenting with intralysosomal storage.^69^ Most recently, trehalose has been found to be endocytically taken up by cells leading to accumulation within the endolysosomal system, resulting in low‐grade lysosomal stress, which in turn stimulates TFEB activation and promotes its nuclear translocation.^172^

##### Genistein

Lysosomal storage diseases (LSDs) are a group of monogenic diseases that result in the progressive accumulation of undegraded material in lysosomes due to the deficiency of specific lysosomal proteins. It is often associated with abnormal intracellular trafficking and an inhibition of the autophagic pathway, resulting in a progressive multisystemic phenotype associated with neurodegeneration.^173^ Mucopolysaccharidosis is an LSD characterized by altered metabolism of glycosaminoglycans (GAGs).^174^ Genistein, a natural isoflavone, has been shown to reduce lysosomal GAGs storage.^175^ Genistein has also been shown to facilitate the nuclear translocation and target gene expression of TFEB, and promote lysosomal synthesis. This suggests that genistein not only involved in the synthesis and degradation of GAGs but also enhances lysosomal function via TFEB.^176^ Niemann‐Pick disease Type C (NPC) is a rare neurodegenerative disease caused by mutations in NPC1 or NPC2 gene which results in an accumulation of cholesterol in lysosomes. Additionally, NPC is also considered to be an autosomal recessive LSD.^177^ Loss of function in NPC1 or NPC2 proteins can causes lysosomal dysfunction and autophagy defects.^178^ A recent study has shown that genistein promotes TFEB translocation to the nucleus and induces autophagy and lysosomal exocytosis, leading to a reduction of cholesterol accumulation in NPC1 patient fibroblasts.^179^ Therefore, genistein may promote the development of novel therapeutic strategies to treat some LSDs.

##### Oleuropein aglycone

Oleuropein aglycone, the major phenolic component found in olive oil, has been shown to fight against neurodegeneration by autophagy activation.^180^ Oleuropein aglycone triggers autophagy in cultured neuroblastoma cells through activation of Ca^2+^‐calmodulin‐dependent kinase kinase β (CaMKKβ)‐AMPK axis and mTOR inhibition.^181^ In transgenic TgCRND8 mice, which overexpressed the Swedish and Indiana mutations in human APP, dietary supplementation of oleuropein aglycone (50 mg/kg of diet) significantly reduced β‐amyloid levels and plaque deposits and improved the cognitive performance in TgCRND8 mice.^182^ However, the authors did not investigate the effect of oleuropein aglycone on TFEB, which may be a potential target for autophagy enhancement.

##### Ouabain

Ouabain, a cardiac glycoside, is a potent inhibitor of the sodium/potassium pump (Na^+^/K^+^‐ATPase).^183^ Ouabain has been reported to inhibit cell growth by blocking the AKT/mTOR signaling pathway.^184^ Ouabain increases the TFEB dephosphorylation and promotes its nuclear translocation, thereby inducing the expression of ALP‐related genes targeted by TFEB. Ouabain also reduces phosphorylated tau through autophagy enhancement, protects against cell damage induced by okadaic acid (OA), improves the rough‐eye phenotype of tau transgenic Drosophila model, and attenuates memory impairment in Tau‐P301L mice.^185^

##### Hep14

HEP14 (5*β*‐O‐angelate‐20‐deoxyingenol), derived from *Euphorbia peplus Linn*, activates TFEB independent of mTORC1, but requires a PKC‐GSK3β cascade. HEP14 directly binds and activates PKC. Furthermore, activated PKC inhibits GSK3β to dephosphorylate and activate TFEB, and subsequently promote the expression of lysosomal genes. Furthermore, HEP14 obviously reduced the accumulation of polyQ‐Htt aggregates in 97Q‐induced HD cell models, promotes lysosome‐dependent clearance of lipid droplets in oleic acid‐induced cells, and reduces Aβ plaques in APP/PS1 mice.^67^

##### Gypenoside XVII

Gypenoside XVII, a major saponin abundant in ginseng and Panax notoginseng, has been shown to release TFEB from TFEB/14‐3‐3 complexes, resulting in TFEB traslocation, rescued autophagic flux, and enhanced lysosomal biogenesis. Gypenoside XVII facilitates the clearance of APP, Aβ40, and Aβ 42 in APP^695swe^ cells and prevents the formation of amyloid plaques in the brain of APP/PS1 mice. In addition, gypenoside XVII attenuates the spatial learning and memory deficits of APP/PS1 mice.^186^

##### Cinnamic acid

Cinnamic acid, a naturally occurring plant‐based product, activates the nuclear hormone receptor PPARα to transcriptionally upregulate TFEB and induce lysosomal biogenesis in mouse primary neurons. Moreover, cinnamic acid treatment in 5xFAD mice remarkably reduce cerebral Aβ plaque burden and improve memory deficit via PPARα.^187^

##### Pseudoginsenoside‐F11

Pseudoginsenoside‐F11(PF11), an ocotillol‐type saponin derived from leaves of *Panax pseudoginseng subsp. himalaicus HARA*, has been shown to be protective against AD both in vivo and in vitro.^188^, ^189^ PF11 facilitates the nuclear translocation of TFEB through mTOR inhibition and improves lysosomal function in oligomeric Aβ‐treated microglial cells. Moreover, PF11 has been proven to increase the degradation of oligomeric Aβ in microglia by improving lysosome function to promote endosome maturation.^190^

##### Fisetin

Fisetin, a flavonol present in numerous fruits and vegetables, has been found to prevent oxidative stress‐mediated neuronal cell death.^191^ Fisetin has been shown to increase the transcriptional activity of TFEB through mTORC1 inhibition, and promote degradation of phosphorylated tau by enhancing autophagy in neurons. In addition, fisetin also reduces levels of phosphorylated tau through the selective autophagy pathway, which is activated by the nuclear factor erythroid 2‐related factor 2 (Nrf2) transcriptional factor.^192^ However, this study has only been verified in vitro. Therefore, it is important to study the role of fisetin in the degradation of endogenous tau via enhanced autophagy in vivo.

##### Paeoniflorin

Treatment of SBMA mice with paeoniflorin, a major component of *Paeonia* plants, induces nuclear translocation of TFEB and enhances mutant AR degradation through NF‐YA upregulation and downstream transactivation of the proteasomal and autophagic proteolytic machinery.^193^

##### Chlorogenic acid

Chlorogenic acid (CGA), a phenolic acid isolated from fruits and vegetables, restores autophagic flux in the brain and alleviates cognitive impairment in APP/PS1 mice by activating the mTOR/TFEB signaling pathway.^194^

##### Qingyangshen

Qingyangshen (QYS), one potential source from traditional Chinese medicine *Cynanchum otophyllum Schneid (Radix Cyanchum Otophylli)*, has received increased attention due to its neuroprotective and anti‐aging activities.^195^, ^196^, ^197^ QYS reduces carboxy‐terminal fragments (CTFs), APP, Aβ, tau aggregates, and improves cognitive function in 3xTg mice through activation of the PPARα‐TFEB pathway and subsequent enhanced ALP pathway.^198^

##### Celastrol

Celastrol is a leptin sensitizer, initially isolated from the roots of *Tripterygium wilfordii*. Recently, a study showed that celastrol reduces phosphorylated Tau aggregates and attenuates cognitive deficits in P301S Tau and 3xTg mice by enhancing TFEB‐mediated autophagy and lysosomal biogenesis. Mechanistically, celastrol promotes TFEB nuclear translocation via mTORC1 inhibition.^199^

#### 5.2.2 Small molecule inhibitors and clinical drugs

##### Rapamycin and its analogs

Rapamycin, a food and drug administration (FDA)‐approved drug, is an inhibitor that interferes with the mTOR signaling pathway, which may be a TFEB agonist.^200^ A number of studies have reported the protective effects of rapamycin and its analogs CCI‐779 in various models of neurodegenerative diseases.^201^ In an animal model of PD induced by intracerebral injection of AAV α‐synuclein, peripheral administration of CCI‐779 efficiently triggered a proautophagic response in the brain via inhibiting mTORC1 activity and increasing the nuclear translocation of TFEB. In addition, CCI‐779 significantly improves motor impairment of PD animals, increases survival of nigral dopamine neurons, maintains the striatal dopamine content, and reduces the accumulation of toxic oligomeric α‐synuclein.^36^ In a Parkin^Q311X^ mutant mouse model, treatment with rapamycin independently restores PGC1α‐TFEB signaling in a manner not requiring parkin E3 ligase activity and abrogates subsequent mitochondrial impairment and age‐related neurodegenerative effects.^122^ Rapamycin has also been proven to effectively reduce Aβ and tau pathology leading to improve cognitive function in several different mouse models of AD.^202^, ^203^, ^204^ However, it is not clear whether rapamycin and its analogs exert neuroprotective effects by regulating TFEB or ALP in these models. Furthermore, more studies are required to fully elucidate this mechanism.

##### Cyclodextrin

The FDA‐approved excipient, 2‐hydroxypropyl‐β‐cyclodextrin (HPβCD), is used to improve the stability and bioavailability of drugs and is widely used as a drug delivery vehicle. HPβCD administration results in activation of TFEB and enhances the cellular autophagic clearance capacity in cells derived from a patient with LSD.^205^ In addition, pharmacological activation of TFEB by HPβCD also enhances autophagic clearance of α‐synuclein aggregates in human neuroglioma cells.^39^ Furthermore, 2‐hydroxypropyl‐γ‐cyclodextrin (HPγCD), another cyclodextrin isoform, enhances autophagy‐lysosomal pathway by increasing TFEB expression and promoting TFEB nuclear translocation. This leads to induced lysosomal biogenesis/autophagy, which plays a crucial function in reducing cholesterol accumulation and cellular stress in NPC1‐deficient cells. In addition, HPγCD also rescues cholesterol accumulation in lysosomes by rescuing the structural abnormalities of lysosomes and promoting lysosome‐ER association.^206^

##### STI‐571

STI‐571, a c‐Abl inhibitor, alleviates MPP^+^‐induced impairment of ALP function by facilitating the nuclear translocation of TFEB, thereby attenuating MPP^+^‐induced cell death in MPP^+^‐treated SN4741 cells and primary midbrain neurons. Under MPP^+^ treatment conditions, c‐Abl directly interacts with GSK3β and phosphorylates GSK3β Tyr216 residue, while STI‐571 reduces the phosphorylation of GSK3β‐Tyr216 induced by MPP^+.^ ^207^

##### Veliparib

Veliparib is a poly(ADP‐ribose) polymerase 1 (PARP1) inhibitor with proven neuroprotective effects in models of PD. In α‐synuclein aggregated cells and mice, veliparib‐induced PARP1 inhibition enhances nuclear transcription of TFEB via SIRT1 mediated downregulation of mTORC1 signaling and also reduces nuclear export of TFEB by attenuating the TFEB‐CRM1 interaction. In addition, oral administration of veliparib prevents neurodegeneration and improves motor activity in PD mouse models.^208^

##### SB203580

SB203580, a p38 inhibitor, significantly improves the motor activity and alleviates abnormal accumulation of α‐synuclein through p38 inhibition in α‐synuclein A53T‐Tg mice. Mechanistically, SB203580 induces chaperone‐mediated autophagy (CMA)‐mediated NLRP3 degradation by activating TFEB‐mediated autophagy by p38 inhibition.^209^

##### Celecoxib

Celecoxib (CXB) is a selective cyclooxygenase‐2 inhibitor, which has significant neuroprotective effects directly on neuron‐like cell types. CXB increases the survival of human dopaminergic cells in 6‐OHDA and paraquat (PQ)‐induced PD models, evidenced by increasing the expression of apolipoprotein D (APOD), TFEB and cathepsin D (CTSD), thereby overcoming the negative effects of neurotoxins on cell survival.^210^

##### Dynasore

Dynasore, a potent inhibitor of dynamin, significantly promotes clearance of protein aggregates formed by mutant polyQ‐Htt protein, but not of damaged mitochondria. Mechanically, dynasore enhances autophagy by repressing the lysosomal localization of mTOR and blocking mTORC1 activity, which in turn enhances TFEB nuclear translocation.^211^

##### Aspirin

Aspirin, one of the most frequently used medications in the world, induces activation of PPARα and upregulates the transcription of TFEB via PPARα in brain cells, which increases lysosomal biogenesis. Accordingly, low‐dose aspirin decreases amyloid plaque pathology in 5xFAD mice in PPARα‐dependent fashion.^212^, ^213^

##### Ibudilast

Ibudilast, a non‐selective inhibitor of phosphodiesterases (PDEs) and an anti‐inflammation drug, significantly enhances clearance of TDP‐43 and SOD1 protein aggregates. Mechanistic investigations have shown that ibudilast can markedly increase TFEB nuclear translocation and induce autophagy and lysosomal biogenesis by targeting mTORC1‐TFEB signaling.^214^

#### 5.2.3 Others

##### Acylated ghrelin

Acylated ghrelin, a gut‐derived neuropeptide, was identified as a natural ligand of the growth hormone secretagogue receptor 1a (GHS‐R1a). Clinical evidence suggests that endogenous acylated ghrelin levels are reduced in PD patients, suggesting a possible link between acylated ghrelin and PD pathogenesis.^215^ Ghrelin enhances autophagic flux and lysosomal biogenesis by upregulating TFEB expression and prompting TFEB nuclear translocation in 6‐OHDA‐induced PD cellular models. Furthermore, ghrelin ameliorates 6‐OHDA‐induced motor dysfunction and protects dopaminergic neurons against 6‐OHDA‐induced neurotoxicity by activating GHS‐R1a, a receptor of ghrelin. However, siRNA knockdown of TFEB could completely abolish the neuroprotective effect of ghrelin.^216^

##### PNU282987

PNU282987, a selective agonist of α7 nAChR, exhibited significant neuroprotective effects against SOD1^G85R^ induced neurotoxicity, which correlates with the activation of autophagy through the AMPK‐mTOR pathway and an increase in intracellular Ca^2+^ influx. Furthermore, PNU282987 promotes the nuclear translocation of TFEB, which in turn enhances lysosomal biogenesis and autophagy activation.^217^

##### F‐SLOH

F‐SLOH, a versatile theranostic agent, can selectively bind to Aβ oligomers with strong fluorescence enhancement and also efficiently crosses the blood–brain barrier (BBB).^218^ Recently, F‐SLOH has been found to significantly reduce the levels of APP, Aβ oligomers, and hyperphosphorylated Tau aggregates in the brains of 5xFAD and 3xTg‐AD mice. In addition, F‐SLOH also significantly ameliorates synaptic deficits and cognitive impairment in AD mouse models. Mechanistic studies have shown that F‐SLOH promote autophagy and lysosomal biogenesis via TFEB dephosphorylation and nuclear accumulation through MAPK1/ERK2 inhibition and PP2A activation.^219^

In addition to some of the small‐molecule compounds described above, a recent study showed that electroacupuncture activates TFEB by inhibiting the AKT‐MAPK1‐mTORC1 pathway and significantly reduces APP and Aβ load and improves cognitive deficits in 5xFAD mice.^220^ Several studies have shown that the natural small‐molecule compounds Mucuna pruriens, Ursolic Acid and Chlorogenic Acid have significant neuroprotective effects on MPTP‐induced dopaminergic neuron damage through their antioxidant and anti‐inflammatory effects in an MPTP‐induced Parkinsonian mouse model, but the mechanism of action is unclear.^221^, ^222^, ^223^ Nrf2 is a critical transcription factor that neutralizes ROS to restore the redox balance of cells, which can be degraded by the Kelch‐like ECH‐associated protein 1 (Keap1), an adapter protein of the Cul3‐ubiquitin E3 ligase complex.^224^ In PD and AD cellular models, the small molecules, myriocin and fisetin, or stimulation of PINK1‐dependent mitophagy are found to be neuroprotective through Nrf2 activation, but the exact mechanism has not been elucidated.^121^, ^192^, ^225^ Moreover, p62 has also been shown to bind to aggregates of ubiquitylated proteins to increase its affinity for Keap1 when phosphorylated at Ser351 in a redox‐independent manner. This results in Keap1 degradation and leaves Nrf2 to translocate in the nucleus to regulate antioxidant and detoxifying genes.^226^ Interestingly, an in vitro study found that TFEB overexpression stabilizes and activates Nrf2 through repression of the Nrf2‐specifc E3 ubiquitin ligase, DDB1 and Cullin4 associated factor 11 (DCAF11). This results in phosphorylation of p62 at Ser349, which further disrupts the binding of Keap1 and Nrf2, thus leading to sustained Nrf2 activation through a positive feedback loop.^227^ This event suggests that TFEB‐mediated positive feedback regulation of p62‐Nrf2 may be a key mechanism linking oxidative stress and autophagy. Furthermore, in PD the above small molecules may mechanistically mitigate oxidative stress‐induced neuronal damage by enhancing the TFEB‐mediated positive feedback regulation of p62‐Nrf2. However, further investigations are required in cellular and animal models.

## 6 CONCLUDING REMARKS

The crucial role of TFEB and its mediated ALP in the clearance of abnormal aggregates has been supported in several preclinical models of neurodegenerative diseases. Several studies have shown that genetic and pharmacological modulation of TFEB are increasingly important strategies for the prevention and treatment of neurodegenerative diseases. However, there are still many uncertainties about whether the small molecules can be used as drug targets for the treatment of neurodegenerative diseases. First, most studies that reported that specific small molecules indirectly activate TFEB by regulating upstream kinases, including mTORC1, PKC, and AKT. However, these kinases also participate in the regulation of several other important cellular biological processes.^228^, ^229^, ^230^ Therefore, whether activation of TFEB by inhibiting or activating these key regulators affects other normal cellular functions needs to be considered and further examined in neurodegenerative diseases models. Secondly, TFEB is also activated by various stressors such as nutrient deficiency, oxidative stress, ER stress, and lysosomal stress, which have been considered as considered risk factors for neurodegenerative diseases. It was necessary to determine whether these investigated small molecules activate TFEB by triggering the cellular stress. Furthermore, it remains unclear whether these small‐molecule TFEB activators can cross the BBB and directly act on brain neurons or glial cells to exert drug effects, which should be carefully determined in animal models. In addition, although acute induction of intracellular protein aggregates degradation can ameliorate some symptoms of disease in several animal models, any long‐term effects of such treatments have not yet been evaluated.

Moreover, as an important intracellular clearance molecule, the level and duration of TFEB activation needs to be tightly regulated. Constitutive activation of MiT/TFE transcription factors, including TFEB, is identified to drive metabolic reprogramming in pancreatic cancer, and upregulation of TFEB can lead to tumorigenesis of renal cell carcinomas.^20^, ^231^ Thus, it is necessary to in‐depth understanding of TFEB functions under various physiological and pathological conditions at the molecular, cellular, and functional levels to further clarify its roles in disease pathology and to inform the development of therapeutics for neurodegenerative diseases.

## AUTHOR CONTRIBUTIONS

F.J. conceptualized the topic. F.J., B.Z., and L.M. drafted the first version of the manuscript. All authors provided content based on their respective expertize, reviewed, and agreed to the final manuscript version.

## FUNDING INFORMATION

This work was supported by the National Natural Science Foundation of China (Grant numbers: 82101340 to FJ), Research Fund for Academician Lin He New Medicine (Grant number: JYHL2019ZD04 to FJ), Shandong Traditional Chinese Medicine Science and Technology Development Plan (Grant number: 2019–0446 to FJ).

## CONFLICT OF INTEREST

The authors declare that there are no competing interests.

## Data Availability

Data sharing is not applicable to this article as no new data were created or analyzed in this study.
